# A novel microscale multilayer PZT MEMS cantilever for resonant photoacoustic gas sensing

**DOI:** 10.1016/j.pacs.2026.100889

**Published:** 2026-10-01

**Authors:** Shan Lin, Yongkang Gong, Aron Michael, Fupeng Wang, Yinghang Jiao, Gang-Ding Peng

**Affiliations:** a School of Electrical Engineering and Telecommunications, University of New South Wales, Sydney, 2052, Australia; b Faculty of Information Science and Engineering, Engineering Research Center of Advanced Marine Physical Instruments and Equipment (Ministry of Education), Ocean University of China, Qingdao, 266100, China

**Keywords:** Photoacoustic spectroscopy, Piezoelectric, PZT thin film, Multilayer MEMS cantilever, Flexural-compliance-adjusted NNEA

## Abstract

This paper reports a novel photoacoustic spectroscopy (PAS) gas sensor based on micro-electro-mechanical systems (MEMS) that achieves resonance matching between a multilayer PZT piezoelectric microcantilever and an H-type acoustic resonator. The device integrates the PZT thin film and exhibits a measured mechanical resonance at 5003.3 Hz with a 3-dB bandwidth of 33.74 Hz. To the best of our knowledge, the proposed device represents one of the most miniaturized thin-film piezoelectric cantilevers reported for PAS gas sensing, with active dimensions of only 1039 μm × 250 μm × 6.43 μm. An H-type photoacoustic cell is designed to align its resonance with the cantilever. Using C_2_H_2_ as a sample gas, the measured results show a linear responsivity of 2.41 μV/ppm over the concentration range of 229.8–5000 ppm, with an excellent linearity of 0.999. Allan deviation analysis of the pure nitrogen background indicates a minimum detection limit (MDL) of 477 ppb at an integration time of 265 s, corresponding to a conventional NNEA of 14.39 × 10^−8^ cm^−1^·W·Hz^−1/2^. A novel metric identified as flexural-compliance-adjusted NNEA (FCA-NNEA) is further introduced to indicate the extent to which the achieved detection performance benefits from the flexural compliance of the cantilever. The proposed device achieves an FCA-NNEA of 0.859 cm^−1^·W·Hz^−1/2^·mm, indicating competitive performance without relying on an extremely compliant cantilever. Together with its microscale dimensions, these results demonstrate a promising route toward compact MEMS PAS sensing.

## 1 Introduction

Trace gas detection is of great importance in environmental monitoring, industrial safety, energy systems, and biomedical diagnostics [[1], [2], [3]]. Among the various sensing techniques, laser spectroscopy has attracted extensive attention because gas molecules exhibit distinct rovibrational absorption fingerprints in the infrared region. Tunable diode laser absorption spectroscopy (TDLAS) is one of the most mature laser-based techniques, benefiting from narrow-bandwidth diode lasers, high selectivity, and fast response [4,5]. However, in direct absorption spectroscopy, weak absorption must be resolved as a small change superimposed on a comparatively large transmitted optical background. Further sensitivity improvement usually requires a long absorption path, which increases optical alignment complexity and system volume [6,7]. Photoacoustic spectroscopy (PAS), by contrast, converts absorbed optical energy into an acoustic pressure. When the target gas molecule transitions are excited by the modulated radiation, non-radiative relaxation produces periodic heating and thermoelastic expansion, thereby generating a pressure wave at the modulation frequency or its harmonics. This pressure wave can be enhanced by an acoustic resonator and detected by a sensitive transducer [8,9]. Since the PAS signal is directly related to the absorption-caused acoustic rather than the small attenuation of the transmitted power, it can substantially suppress the large transmitted-intensity background and provide high sensitivity within a compact interaction volume. The acoustic detector is also not intrinsically wavelength-selective, allowing a common transducer architecture to be used with different excitation wavelengths.

In the PAS system, the acoustic transducer is a critical component because the absorption-induced pressure wave is usually very weak. Conventional condenser or electret microphones have been widely used because of their mature fabrication and convenient readout. Nevertheless, the relatively large packaging volume of these microphones limits further miniaturization and compact optical-acoustic integration [10]. With the development of micro-electro-mechanical systems (MEMS), silicon-based MEMS microphones reduce the device size and are attractive for integrated PAS systems. However, the resonance and responsivity of MEMS microphones depend on gas damping, while thermomechanical and electronic noise can limit their ultimate performance [11,12]. Quartz-enhanced photoacoustic spectroscopy (QEPAS) addresses part of this limitation by using the quartz tuning fork (QTF) as a compact piezoelectric resonant detector. The high-quality factor and narrow resonance bandwidth of QTFs enable strong rejection of environmental acoustic noise [[13], [14], [15]]. However, standard commercial QTFs typically resonate at 32.768 kHz, which can be too high for molecules with slow vibrational-translational relaxation at atmospheric pressure. Recent studies have explored custom-designed QTFs with resonance frequencies reduced from approximately 12.45 kHz down to below 3 kHz [[16], [17], [18]]. These developments have substantially broadened the range of sensing QTFs available, allowing the detection frequency to better accommodate relatively slow molecular relaxation without relying on the addition of auxiliary gases to modify the relaxation rate of the target species. Nevertheless, the intrinsically low piezoelectric coefficient of quartz still constrains electromechanical conversion efficiency and limits further sensitivity enhancement. To overcome this material limitation and strengthen strain-to-charge transduction, tuning forks have been fabricated from materials with higher piezoelectric coefficients. Lithium niobate provides stronger piezoelectric coupling while retaining mechanical and thermal stability, and PZT ceramics offer an even stronger piezoelectric response, enabling a larger electrical output for a given mechanical deformation [19,20]. Recent tuning fork-based laser spectroscopy has also explored annular forks, heterodyne multiplexing, orthogonal mode decoupling, acoustic coupling, cascaded resonators, and data-driven denoising [[21], [22], [23], [24], [25], [26]]. In parallel, dual-frequency acoustic cells have been developed for simultaneous multi-gas PAS measurements [27]. Collectively, these studies demonstrate that the performance of tuning-fork transducers can be extended through coordinated optimization of piezoelectric material, structural geometry, and signal processing.

Within this broader landscape, cantilever transducers provide a complementary route to miniaturized acoustic detection. Their single-ended compliant geometry offers considerable flexibility in tailoring the resonance frequency and flexural rigidity through the beam dimensions and multilayer configuration. These characteristics enable cantilever transducers to achieve a strong mechanical response to weak pressure excitation and to support either optical displacement measurement or integrated piezoelectric readout. Silicon cantilevers combined with optical interferometric readout have demonstrated high displacement sensitivity and low sample-volume requirements [28,29]. Recent silicon-MEMS studies have investigated optimized geometries and vibrational modes from approximately 1–10 kHz, achieving a system-level N_2_O detection limit of 15 ppb near 10 kHz with a 100-mHz detection bandwidth [30]. Dual-tube resonator integration has enabled a reported detection limit of 0.34 ppb at 10 s, while combined optical and acoustic enhancement with a racket-shaped silicon cantilever has achieved 17 ppt N_2_O detection at 20 s [31,32]. Long-averaging systems have even reached a noise equivalent concentration of 650 parts-per-quadrillion (ppq) for HF at 32 min [33]. Optical fiber cantilever PAS systems have further explored non-contact acoustic coupling, pressure-adaptive sensing, and enhanced acoustic-cavity configurations, extending cantilever-based PAS toward more diverse operating environments and system architectures [[34], [35], [36], [37]]. However, optical readout requires additional interferometric components and precise alignment, which increases system complexity and may limit field-deployable integration. Piezoelectric cantilevers can directly convert pressure-induced bending into electrical signals, simplifying the detection configuration. Polyvinylidene fluoride (PVDF) film has been explored for direct electrical readout in PAS because of the low stiffness, mechanical flexibility, and convenient film processing [38]. However, its charge generation and electromechanical conversion efficiency are generally lower than those of lithium niobate and PZT materials, and polymer loss can affect resonant operation. Lithium niobate offers stronger piezoelectric coupling and opportunities for photonic integration, whereas PZT provides comparatively large transverse piezoelectric coefficients and strong electromechanical coupling and can be deposited as a MEMS-compatible thin film. Ledermann et al. developed a PZT thin-film cantilever for miniaturized photoacoustic detection [39]. The PZT thin film is selected primarily to strengthen strain-to-electrical transduction, but the design mainly operates between approximately 5 and 100 Hz and is not designed for kilohertz acoustic resonance. Therefore, a microscale piezoelectric transducer that combines efficient electromechanical conversion with a kilohertz mechanical resonance remains desirable for PAS.

In this work, a novel multilayer PZT MEMS cantilever with active dimensions of only 1039 μm × 250 μm × 6.43 μm is designed, fabricated, and integrated with an H-type photoacoustic cell co-designed for operation near 5 kHz. The bare cantilever exhibits a measured mechanical resonance at 5003.3 Hz with a 3-dB bandwidth of 33.74 Hz, whereas the assembled PAS system exhibits a response maximum at 4988 Hz. For C_2_H_2_ sensing at 1531.58 nm, a linear fit over the experimentally tested concentration range of 229.8–5000 ppm achieves a responsivity of 2.41 μV/ppm (R^2^ = 0.999). Under pure N_2_ background, the Allan deviation gives a minimum detection limit of 477 ppb at an integration time of 265 s. Using the measured incident optical power of 30 mW, the corresponding verified normalized noise equivalent absorption (NNEA) is 14.39 × 10^−8^ cm^−1^·W·Hz^−1/2^. Furthermore, a flexural-compliance adjustment to the conventional NNEA is introduced to account for the influence of cantilever geometry and mechanical compliance, providing a basis for comparing cantilever-based photoacoustic sensors.

## 2 Sensor design and characterization

### 2.1 Operating principle and integrated configuration

Fig. 1 summarizes the general principle of piezoelectric cantilever resonance photoacoustic spectroscopy and the integrated sensor developed in this work. As illustrated in Fig. 1(a), wavelength-modulated laser radiation is absorbed by the target gas molecules, and the periodic absorption followed by non-radiative relaxation generates the photoacoustic pressure field. When the photoacoustic excitation frequency *f*_PA_ is matched to the mechanical resonance *f*_cantilever_ of the cantilever, the flexural vibration is driven to the maximum by the photoacoustic pressure, and the resulting strain is converted by the piezoelectric layer into an electrical signal. Based on this transduction process, the proposed integrated sensor is shown in Fig. 1(b). The H-type photoacoustic cell serves as both the gas chamber and the acoustic resonator and is integrated with a fiber collimator, gas inlet, and outlet ports. The collimated laser beam propagates along the cell, while the absorption-induced pressure is resonantly enhanced in the central cavity and coupled through a pressure outlet at the cavity center to the upper surface of the cantilever, as highlighted in the enlarged view. The resulting bending vibration is converted into a piezoelectric signal and routed through the electrical outlet for subsequent amplification and demodulation. The cantilever module is secured to the gas cell using screw tightening. To prevent acoustic leakage and maintain gas tightness, O-rings are installed at the contact interfaces, and hot-melt adhesive is used to seal the other connection ports.

**Fig. 1:**
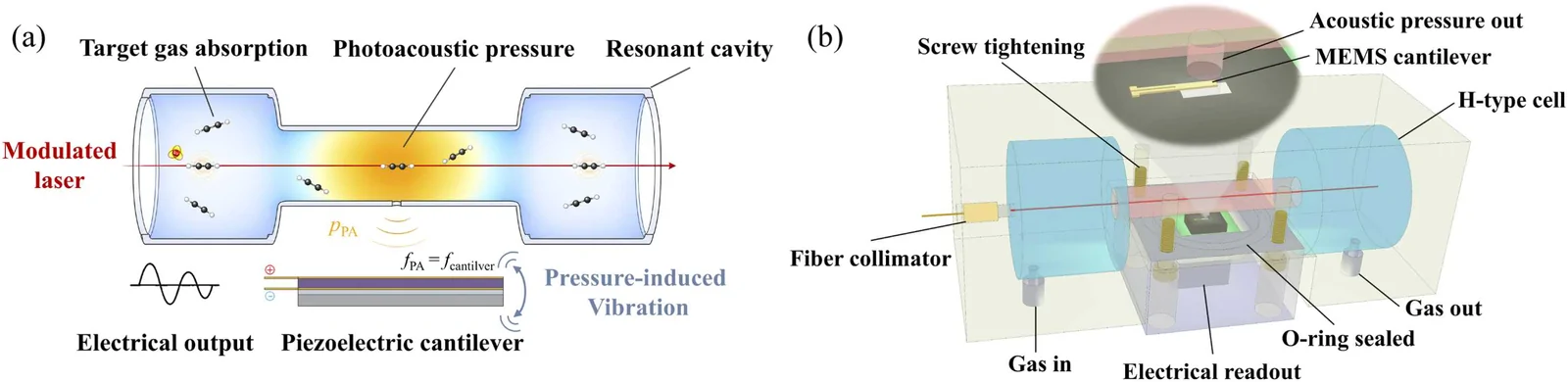
(a) Transduction process in piezoelectric cantilever resonance photoacoustic spectroscopy. (b) Schematic of the proposed MEMS PZT cantilever-based photoacoustic sensor.

### 2.2 MEMS PZT cantilever design, fabrication, and characterization

#### 2.2.1 Cantilever design and finite-element analysis

As the core photoacoustic transducer in the proposed photoacoustic sensor, the designed multilayer cantilever is actuated by a PZT layer interposed between two Pt/Ti electrode layers, which are situated on SiO_2_ and Ultra-High Vacuum E-beam Evaporated (UHVEE) polysilicon layers serving as passive structural components. As shown in Fig. 2, the dimensions refer to the suspended rectangular section with an effective length of 1039 μm and a width of 250 μm. The rear part of the structure is fixed to the substrate and therefore does not participate in the flexural vibration. From top to bottom, the thicknesses of different layers are 0.115 μm, 1.2 μm, 0.115 μm, 1 μm, and 4 μm, respectively. To evaluate whether the designed multilayer MEMS PZT cantilever has a suitable mechanical resonance for photoacoustic detection, finite-element simulations were performed using COMSOL Multiphysics, and only the active rectangular section was retained in the simulations. A three-dimensional multilayer model was established based on the designed geometry and material parameters, with one substrate side fixed and all other surfaces mechanically free. The solid mechanics and electrostatics interfaces were coupled through the piezoelectric effect module, with the PZT layer defined as the active piezoelectric domain. A coupled eigenfrequency analysis was performed to determine the first four eigenmodes. The stress and piezoelectric surface-charge distributions of the fundamental mode were subsequently evaluated. As shown in Fig. 3, the fundamental mode occurs at 5124.7 Hz and exhibits an out-of-plane bending pattern, with the displacement increasing continuously from the clamped end to the free end. The second flexural mode at 31.582 kHz contains an internal nodal region along the beam length, whereas the mode at 42.544 kHz is predominantly torsional, with opposite transverse motion across the cantilever width. The mode at 88.443 kHz exhibits a higher-order flexural pattern with multiple nodal regions. The fundamental mode was selected because its node-free bending pattern enables effective coupling to the normal acoustic pressure and avoids the partial charge cancellation associated with the out-of-phase deformation of higher modes. Based on these results, the stress and piezoelectric charge distributions of the fundamental mode were further analyzed to evaluate its electromechanical conversion characteristics, as shown in Fig. 4.

**Fig. 2:**
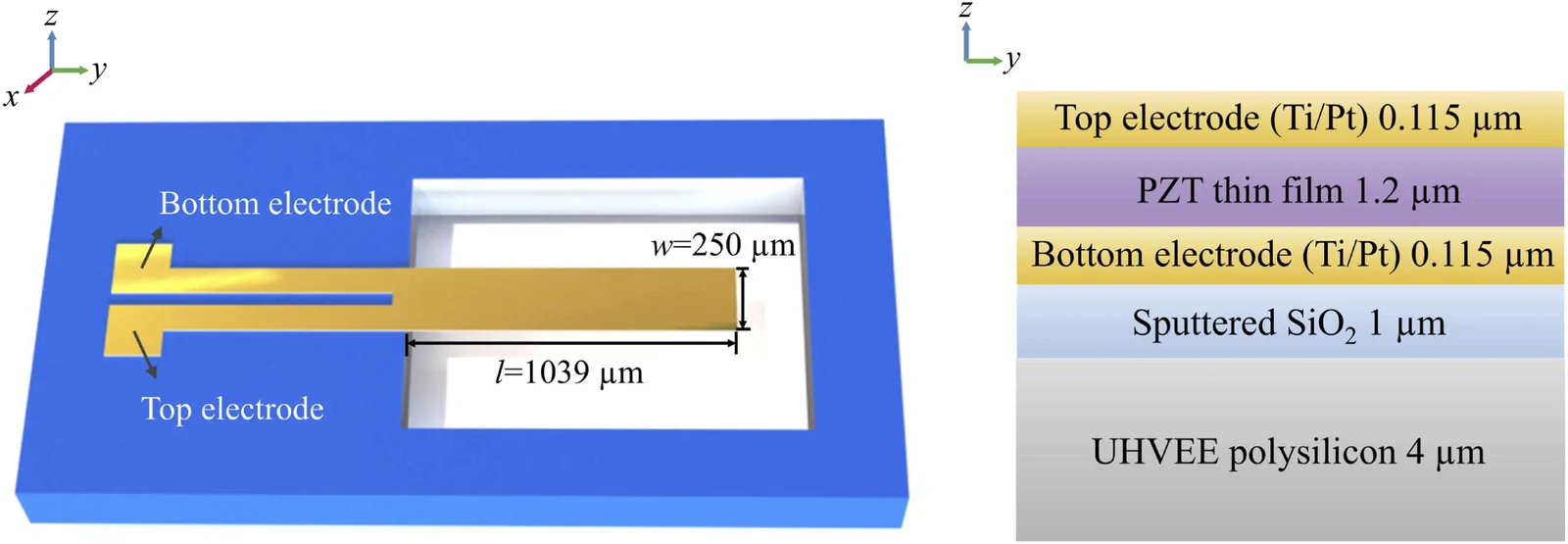
Geometry and multilayer structure of the MEMS PZT cantilever.

**Fig. 3:**
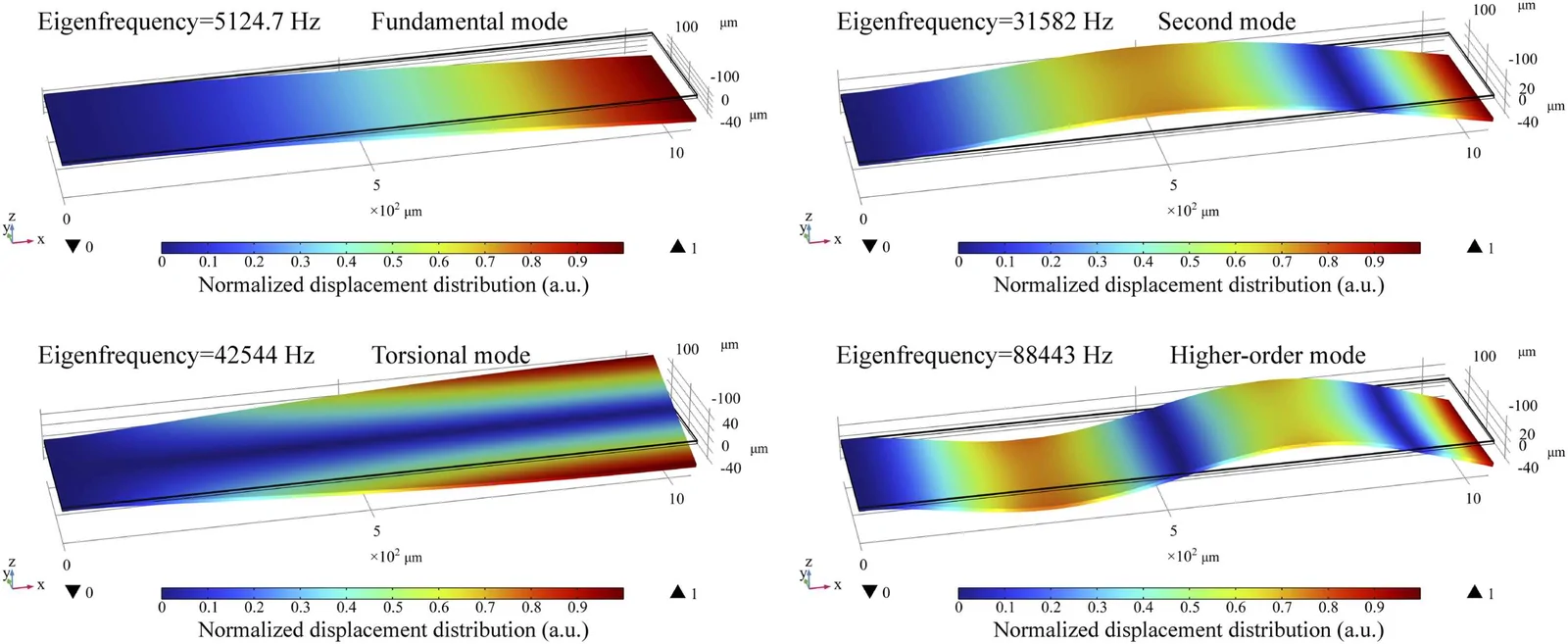
Simulated eigenmodes of the multilayer MEMS PZT cantilever.

**Fig. 4:**
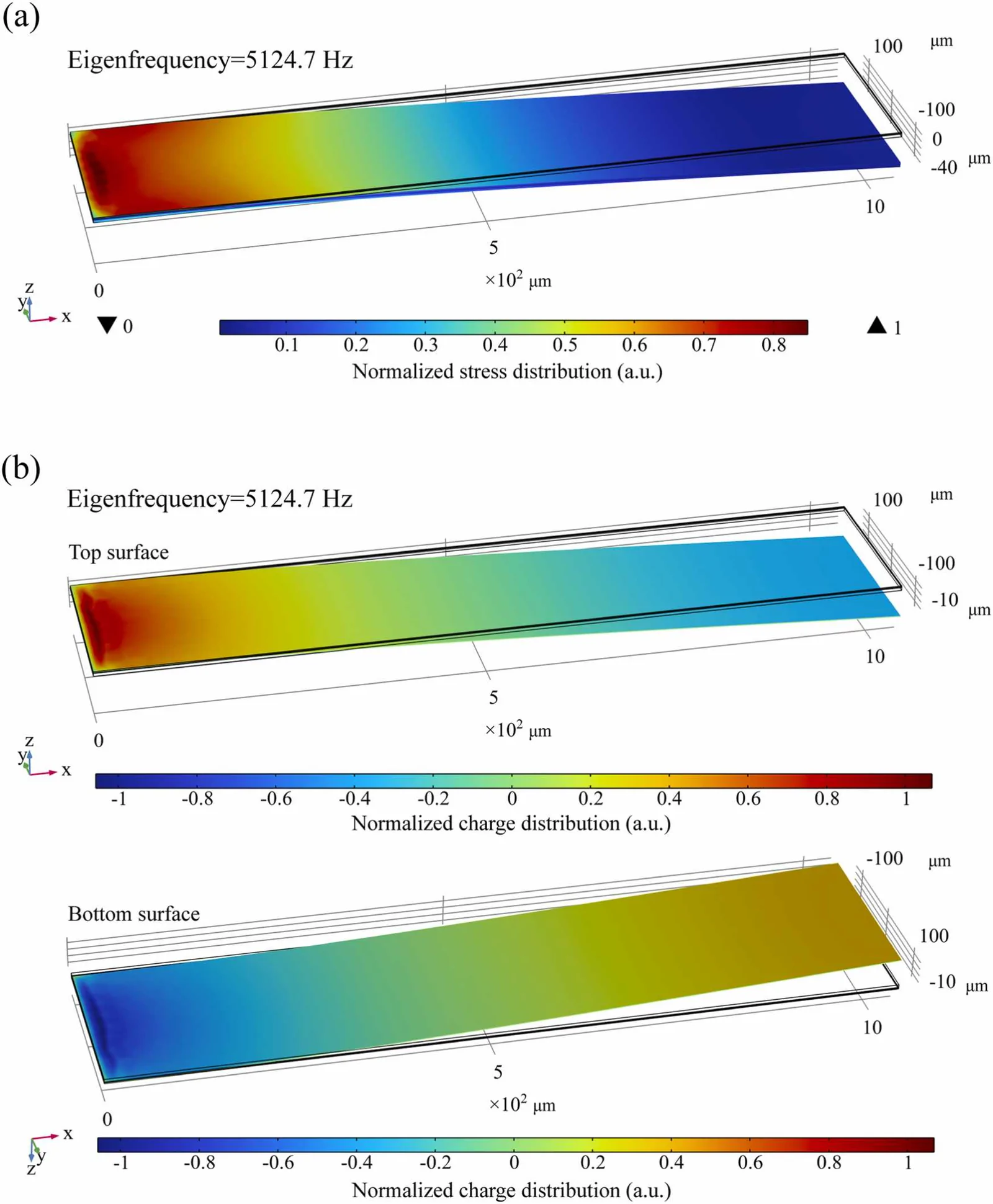
Coupled electromechanical response of the fundamental flexural mode at 5124.7 Hz. (a) Normalized stress distribution. (b) Normalized charge distributions on the top and bottom surfaces of the PZT layer.

As shown in Fig. 4(a), the bending stress is concentrated near the clamped end and gradually decreases toward the free end. The charge distributions in Fig. 4(b) follow the same spatial trend, indicating that the dominant piezoelectric charge is generated in the high-stress region near the clamped end. The dominant charge near the clamped end is positive on the top surface of the PZT layer and negative on the bottom surface. This opposite-polarity distribution is consistent with the PZT poling direction and the electrode configuration, in which the top electrode is defined as the positive signal terminal and the bottom electrode as the negative terminal. The predicted fundamental frequency near 5 kHz is suitable for kHz-level resonant PAS operation, while the stress and charge density confirm the intended conversion of resonant bending into an electrical output.

#### 2.2.2 Fabrication of the multilayer PZT cantilever

Following the simulation, the cantilever was fabricated on a polysilicon-on-insulator (POI) wafer utilizing a standard sequence of deposition, patterning, etching, and release processes. The process started from a silicon wafer substrate with a 1 µm thick thermally grown SiO_2_ on which a 4-μm-thick UHVEE polysilicon structural layer was deposited. Following that, an approximately 1 µm SiO_2_ film was sputtered on the top of the polysilicon layer using RF sputtering and then thermally treated at 700 °C, yielding a tailored compressive stress of 150 MPa. The piezoelectric stack was then formed by sequentially depositing a 0.115 µm Ti/Pt bottom electrode, a 1.2 µm active piezoelectric layer from a sol-gel PZT solution through several repeated spin-dry-crystallize cycles, and a corresponding 0.115 µm Ti/Pt top electrode (Fig. 5(a)).

**Fig. 5:**
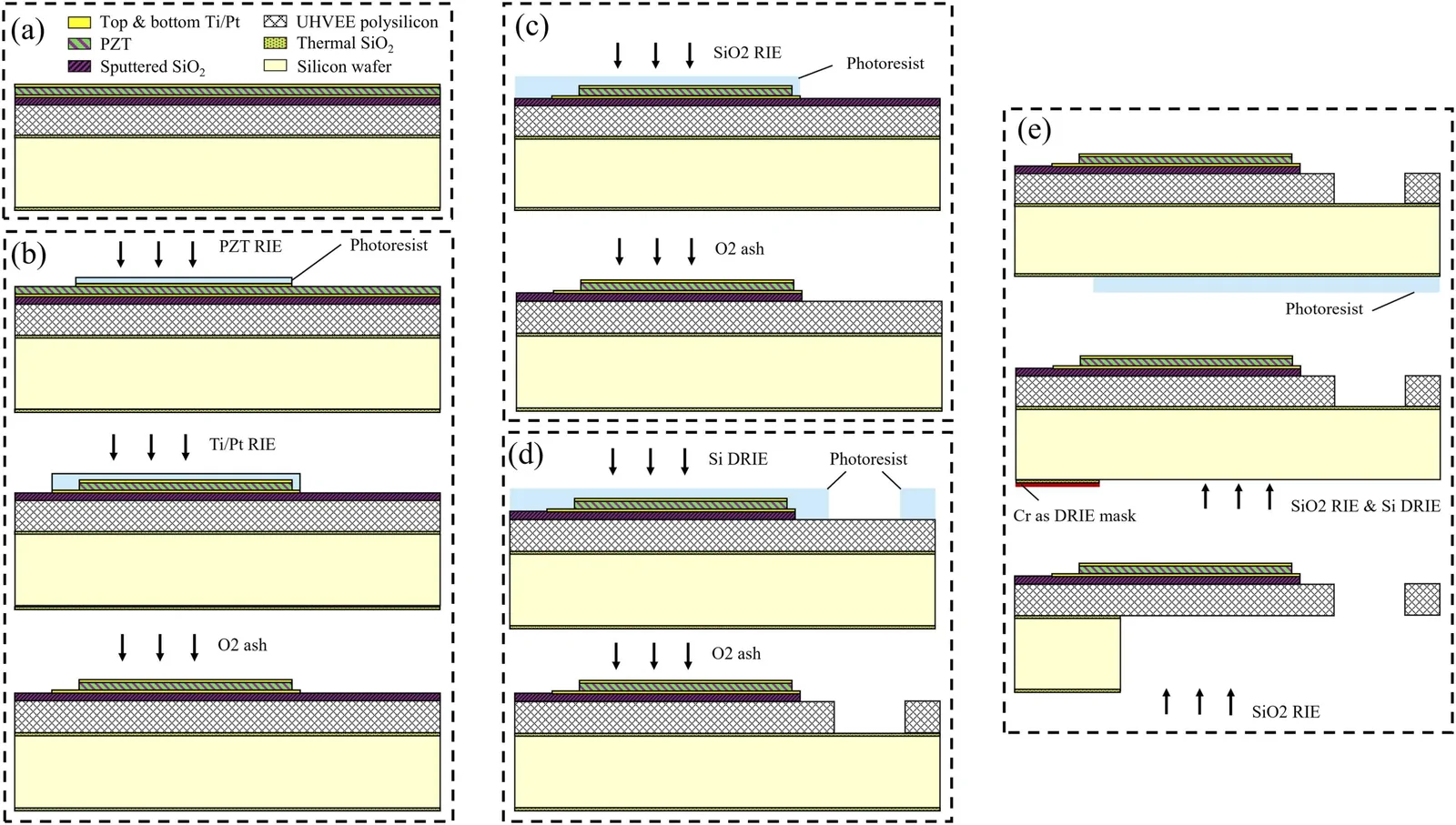
Fabrication process of multilayer MEMS cantilever.

The device geometry was precisely defined through successive photolithography and reactive ion etching (RIE) steps. Specifically, the top electrode and the PZT layer were etched using a C_4_F_8_/He plasma, followed by patterning the bottom electrode with thick photoresist as the etch mask, which was subsequently removed by O_2_ ash (Fig. 5(b)). The exposed sputtered SiO_2_ was then patterned and etched using a photoresist as an etch mask, which was removed by O_2_ ash (Fig. 5(c)). The structural profile of the microcantilever was transferred onto the underlying UHVEE polysilicon layer utilizing a patterned photoresist as a mask and standard Bosch process (Si RIE), stopping precisely at the thermal SiO_2_ boundary. The photoresist was then removed by O_2_ ash (Fig. 5(d)). Following Cr lift-off on the backside, the microcantilever was ultimately released by sequential RIE of the thermal SiO_2_ on the backside, deep reactive ion etching (DRIE) of the bulk silicon, and RIE of the thermal oxide on the front side (Fig. 5(e)).

#### 2.2.3 Morphological and dynamic characterization

After fabrication, the scanning electron microscopy (SEM) image of the released MEMS cantilever is shown in Fig. 6. The dynamic response of the fabricated cantilever was measured using a Polytec Microsystem Analyzer MSA-500. During the mechanical characterization, the cantilever was not coupled to the photoacoustic cell and was driven electrically by applying a swept sinusoidal voltage between the top and bottom electrodes. The excitation frequency was swept from 4000 to 6000 Hz, and the out-of-plane vibration was measured near the free end using the laser Doppler vibrometer of the Polytec MSA-500 at room temperature and atmospheric pressure. As shown in Fig. 7(a), the fabricated cantilever exhibits a clear fundamental resonance near 5 kHz. The enlarged response around the peak in Fig. 7(b) was fitted with a Lorentzian function, yielding a resonance frequency of 5003.3 Hz and a 3-dB bandwidth of 33.74 Hz. The corresponding quality factor is approximately 148. The small frequency shift of the measured resonance can be attributed to fabrication tolerances, residual stress, and material-property variations. The close agreement confirms the successful fabrication of the multilayer PZT cantilever and verifies that its dynamic properties are suitable for integration with the acoustic resonator.

**Fig. 6:**
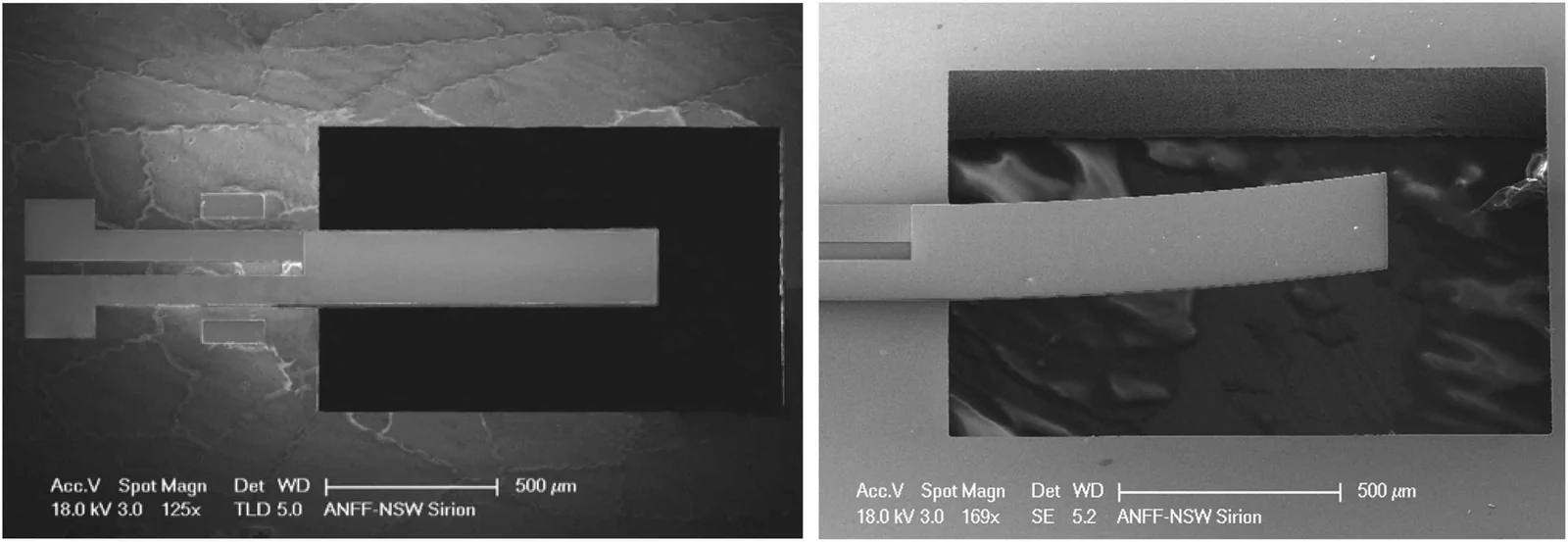
SEM images of the released cantilever beam.

**Fig. 7:**
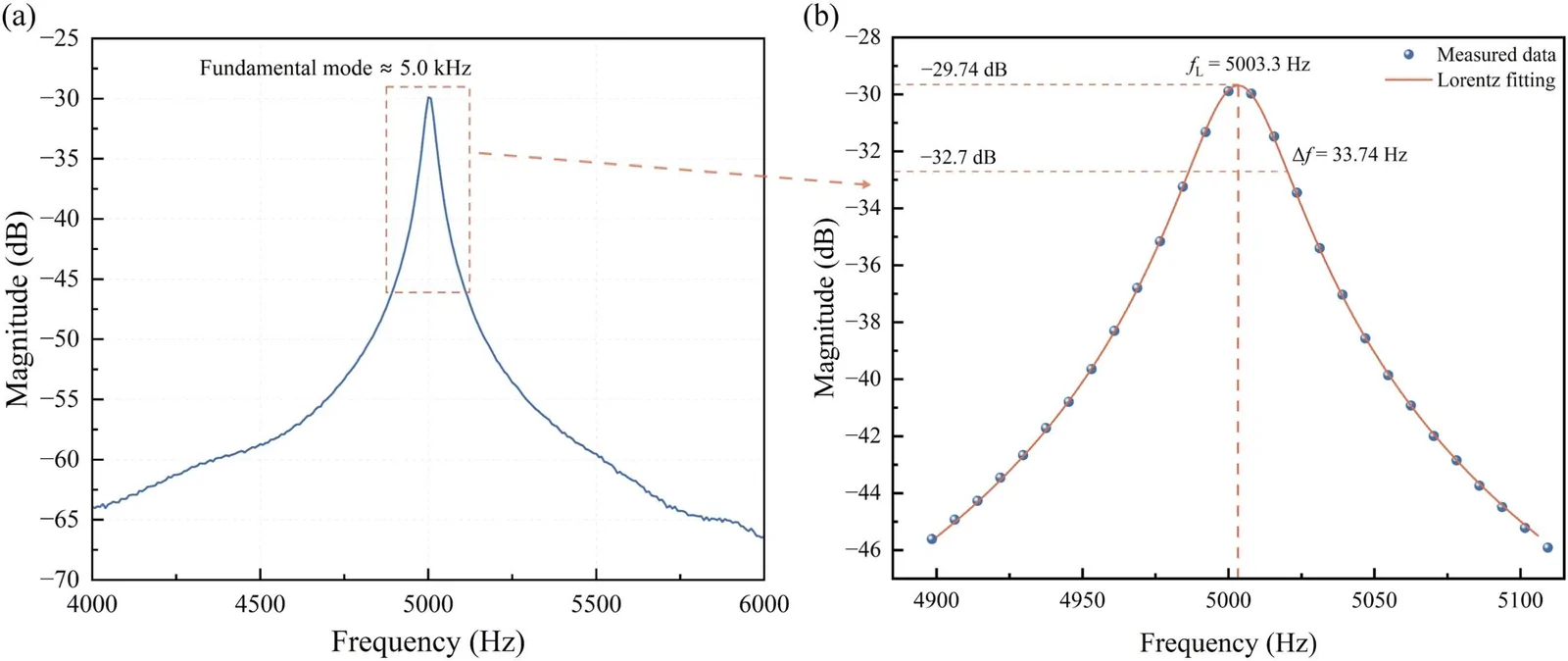
Dynamic characterization of the fabricated cantilever. (a) Measured vibration magnitude around the fundamental mode in ambient laboratory air at room temperature. (b) Enlarged resonance peak with Lorentz fitting and 3-dB bandwidth analysis, yielding *f*_L_ = 5003.3 Hz and Δ*f* = 33.74 Hz.

### 2.3 Design of the H-type photoacoustic cell

To enhance the photoacoustic pressure and coordinate the acoustic resonance with the dynamic response of the fabricated cantilever, an H-type photoacoustic cell was designed, as shown in Fig. 8(a). The cell consists of a central cylindrical resonant cavity connected to two enlarged buffer cavities. The main cavity has a length of 31.2 mm and a diameter of 5 mm, while each buffer cavity has a length of 15.8 mm and a diameter of 21.4 mm. The enlarged buffer cavities reduce the influence of gas-flow disturbances and external acoustic noise on the central resonant region. An acoustic pressure outlet is located at the center of the main cavity, thereby enabling the enhanced acoustic pressure to be coupled to the upper surface of the MEMS cantilever.

**Fig. 8:**
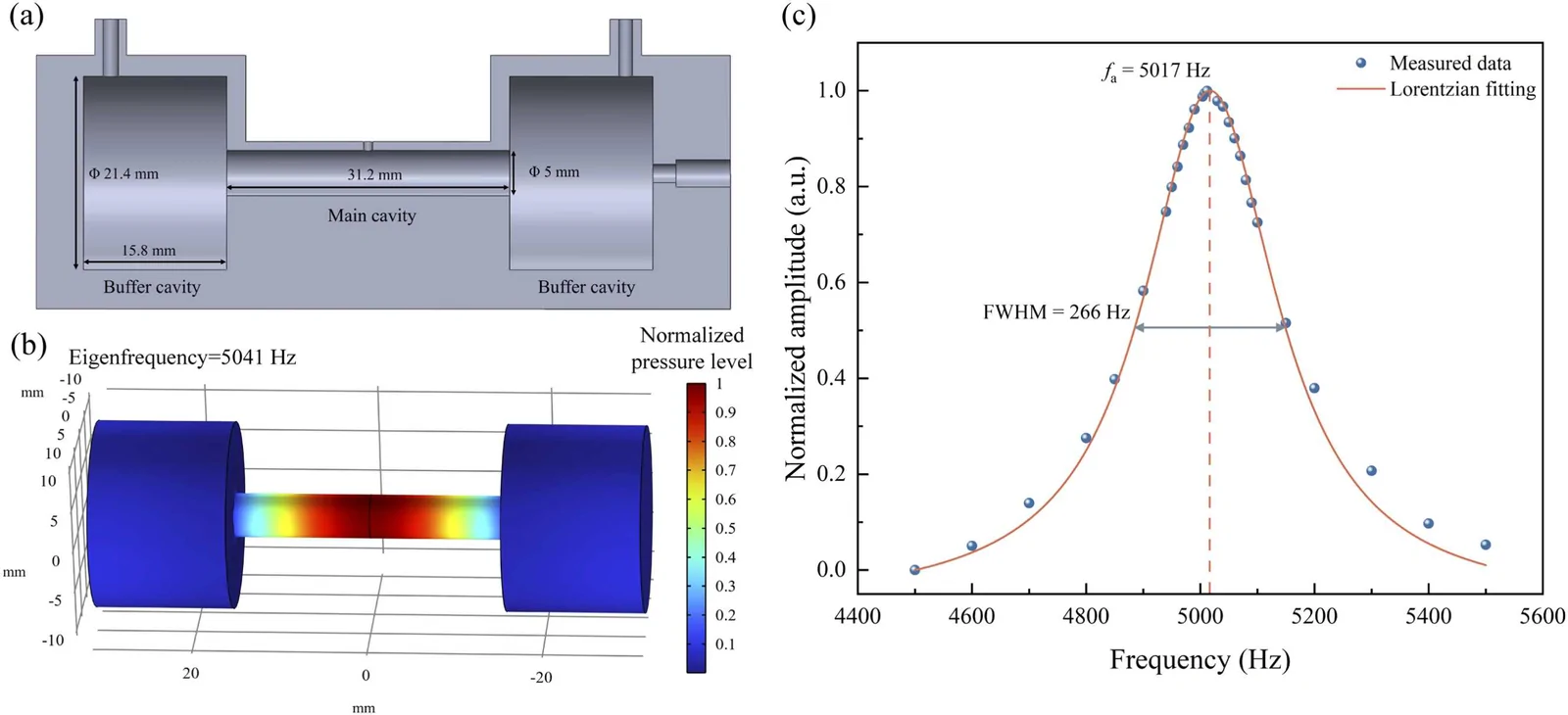
Design, simulation, and characterization of the H-type photoacoustic cell. (a) Geometrical configuration. (b) Simulated eigenfrequency and normalized pressure distribution. (c) Measured frequency response of the cell.

A finite-element model was established to evaluate the eigenfrequency and pressure distribution of the designed cell. As shown in Fig. 8(b), the selected mode exhibits a strong pressure excitation around the center of the main cavity, whereas the amplitude decreases substantially within the two buffer cavities. The simulated eigenfrequency is 5041 Hz, which is close to the measured cantilever resonance of 5003.3 Hz. The resonant cavity was then 3D-printed using resin material through stereolithography (SLA) with printing precision of 0.1 mm. The bare printed photoacoustic cell was then characterized by photoacoustic excitation sweeping, and the acoustic amplitude was recorded with a reference microphone. As shown in Fig. 8(c), the measured response is described by a Lorentzian profile, yielding a peak resonance frequency of 5017 Hz. The experimental result is well aligned with the simulation. The small frequency difference between these two is attributed to dimensional variations during the 3D-printing that are not fully represented in the idealized model. The measured cell response has an FWHM of 266 Hz, which is substantially broader than the resonance bandwidth of the bare cantilever. Therefore, the independently measured resonance frequencies of the cantilever (5003.3 Hz) and the acoustic cell (5017 Hz) indicate an approximate resonance match.

## 3 Experimental setup and gas-sensing performance

### 3.1 Gas-sensing experimental setup

To evaluate the capability of the designed MEMS PZT cantilever for photoacoustic gas detection, an experimental system was set up as shown in Fig. 9. A fiber-coupled distributed feedback (DFB) laser (S/N:5230240, Optpchip optpelectronics Ltd) with a center wavelength of 1531.8 nm was employed to target the C_2_H_2_ absorption line at 1531.58 nm (6529.17 cm^−1^), and the laser output was coupled into the 3D-printed photoacoustic cell by a fiber collimator. As shown in Fig. 10 (a), based on the HITRAN database, the selected C_2_H_2_ line is centered at 6529.17 cm^−1^ (1531.59 nm) with a line strength of 1.165 × 10^−20^ cm^−1^/(molecule·cm^−2^), which is approximately three to four orders of magnitude stronger than the interfering species, including CO_2_, H_2_O, and CH_4_. The simulated absorbance spectral region from 6528 to 6536 cm^−1^ is enlarged in Fig. 10(b) to compare the two strong C_2_H_2_ absorption lines. Although the absorbance at 6534.36 cm^−1^ is slightly stronger, it is more susceptible to interference from water vapor under high-humidity conditions. In contrast, the selected line in this work at 6529.17 cm^−1^ combines strong absorption with good spectral separation from the H_2_O and CO_2_ backgrounds.

**Fig. 9:**
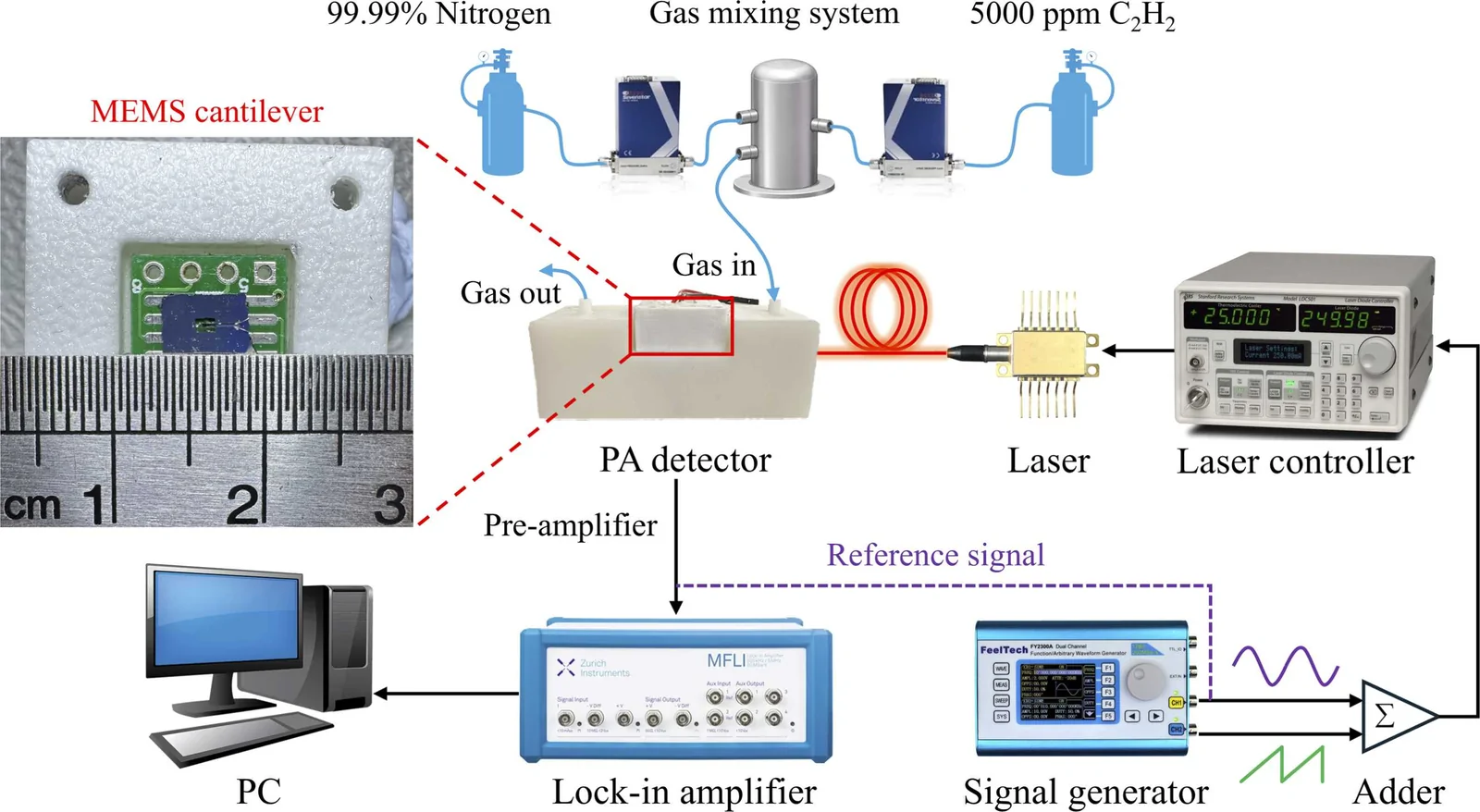
Experimental setup for the MEMS PZT cantilever-based photoacoustic system.

**Fig. 10:**
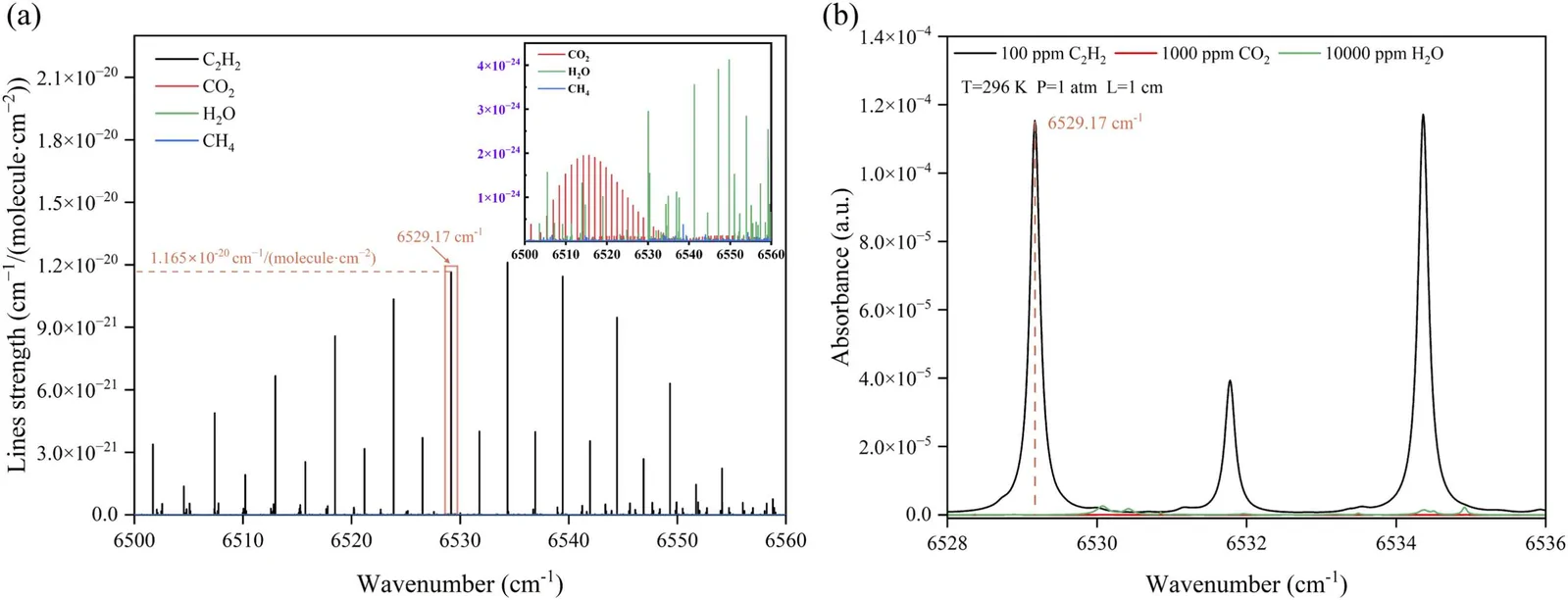
Spectroscopic selection of the C_2_H_2_ absorption line based on the HITRAN database. (a) Line strengths of C_2_H_2_ and the main potential interfering species over 6500–6560 cm^−1^. The inset shows the weaker CO_2_, H_2_O, and CH_4_ lines on an enlarged scale. (b) Enlarged comparison of the simulated absorbance over 6528–6536 cm^−1^.

A sawtooth wave at 0.1 Hz with a peak amplitude of 1 V was used to scan the laser wavelength, while a sinusoidal waveform at a high frequency was added for wavelength modulation. Both signals were generated by a dual-channel function generator and applied to the analog modulation input of a laser controller (LDC501, Stanford Research Systems), which was operated at a gain of 50 mA/V to drive the DFB laser. The sinusoidal signal was also connected to a lock-in amplifier (MFLI, Zurich Instruments) as a reference. The laser temperature was stabilized at 28 ℃ by the laser driver as well. The piezoelectric signal from the cantilever was first amplified by a laboratory-built readout circuit consisting of two OPA2140 amplifiers. The first stage converts the piezoelectric charge into voltage using a 1 MΩ feedback resistor in parallel with a 10 pF capacitor, while the second stage provides a nominal voltage gain of 100 using 1 kΩ input and 100 kΩ feedback resistors. The circuit is powered by ±5 V supplies, and its output is connected to the lock-in amplifier. The demodulated second harmonic (2 f) signal was recorded by a personal computer, and its central peak amplitude was extracted for concentration evaluation. Gas mixtures with different C_2_H_2_ concentrations were prepared by a gas mixing system. High-purity nitrogen was used as the background, while 0.5% C_2_H_2_ was used as the sample gas source. All standard gases used were dry without additional humidification. By adjusting the flow rates of the two gases, C_2_H_2_ with different concentrations was introduced into the cell for measurement. All the experiments were carried out at room temperature and atmospheric pressure.

### 3.2 Optimization of modulation parameters

Since wavelength modulation spectroscopy was employed and the photoacoustic signal was demodulated at the second harmonic, the acoustic excitation frequency was twice the laser modulation frequency. Therefore, the modulation frequency was first optimized according to the actual frequency response of the complete PAS system. A 2000 ppm C_2_H_2_ sample was introduced into the photoacoustic cell, and the laser modulation frequency was changed from 2465 Hz to 2525 Hz, corresponding to a 2 f frequency range of 4930–5050 Hz. At each modulation frequency, the peak amplitude of the detected 2 f signal was recorded to obtain the system frequency response. As shown in Fig. 11(a), the measured response exhibits a clear resonance peak at 4988 Hz, and the full width at half maximum (FWHM) was determined to be 39.4 Hz with Lorentzian fitting. Compared with the resonances of the bare cantilever and photoacoustic cell, the assembled system exhibits a slightly lower resonance frequency. The bare cantilever was characterized in an open atmospheric environment, whereas after integration it interacts with the confined gas volume and acoustic pressure field at the outlet of the photoacoustic cell. The resulting gas loading, together with the modified acoustic boundary conditions and effective impedance, changes the dynamic response of the integrated system. Minor variations introduced by mounting and sealing may also contribute to the frequency shift. Nevertheless, the assembled resonance remains within the relatively broad resonance band of the bare cell, thereby maintaining effective resonance matching. Then the laser modulation frequency was set to 2494 Hz in the subsequent experiments, so that the generated 2 f photoacoustic signal matched the resonance frequency of the MEMS cantilever-based PAS sensor. The modulation amplitude of the high-frequency sinusoidal signal applied to the laser driver was further optimized to maximize the 2 f signal. As shown in Fig. 11(b), the normalized amplitude initially increases with the modulation current, reaches its maximum at 16.25 mA, and then decreases slightly at higher modulation amplitudes. Therefore, this modulation depth was selected for the subsequent detection.

**Fig. 11:**
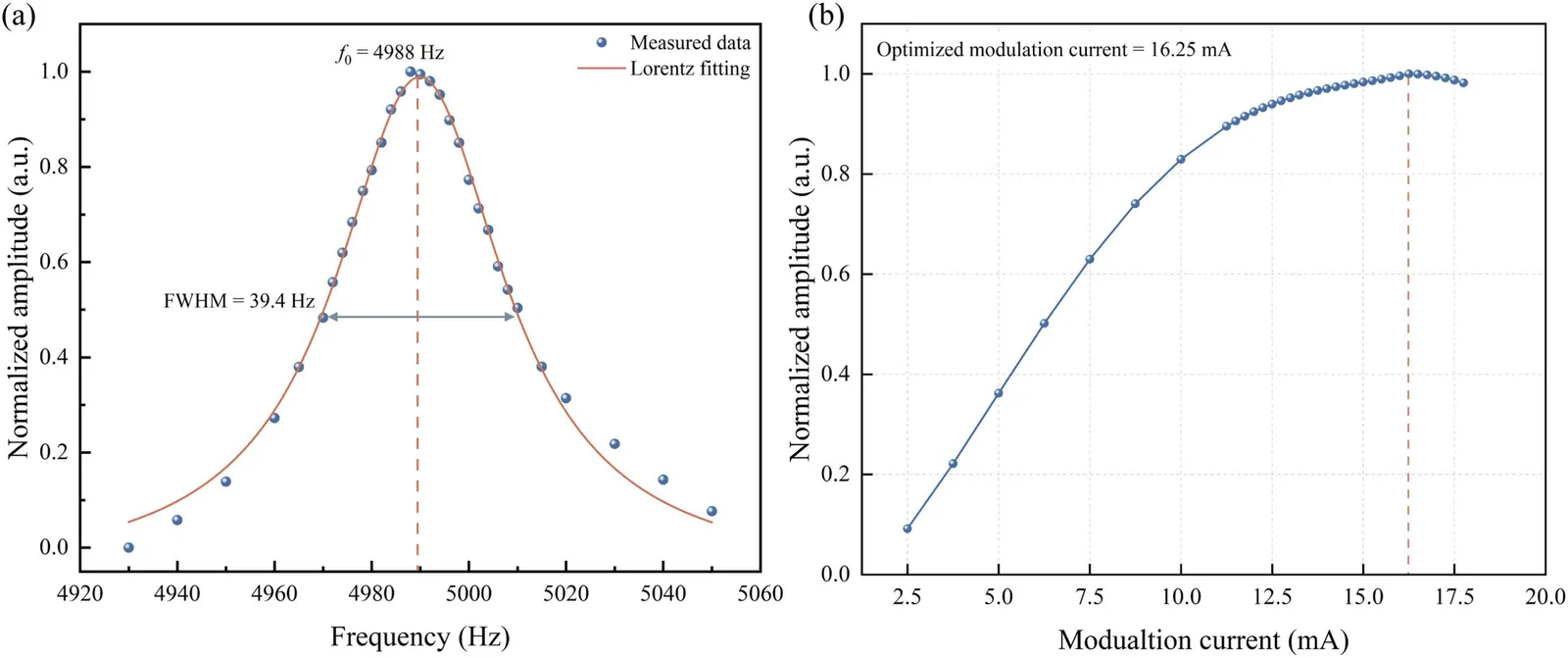
Optimization of the laser modulation parameters. (a) Measured frequency response of the complete PAS system. (b) Optimal modulation depth of the system.

### 3.3 C_2_H_2_ sensing performance

After optimizing the modulation frequency and modulation amplitude, the detection performance of the MEMS cantilever-based PAS system was evaluated. Different C_2_H_2_ concentrations from 229.8 to 5000 ppm were prepared using the gas mixing system, and the 2 f photoacoustic signals were recorded as shown in Fig. 12(a). The peak values of the 2 f signals were further extracted, and linear fitting was performed as shown in Fig. 12(b). This resulted in a concentration responsivity of 2.41 μV/ppm and an R^2^ of 0.999, indicating excellent linearity and confirming the feasibility of using the MEMS PZT cantilever as a sensing element. To evaluate the detection limit, the background signal was recorded for approximately 0.5 h while pure nitrogen flowed through the photoacoustic cell. As shown in Fig. 13(a), the standard deviation (1σ) was calculated to be 12.76 μV. Allan deviation analysis was subsequently performed on the measured noise data. As shown in Fig. 13(b), the detection limit was improved to 1.07 ppm at 100 s integration time. The minimum detection limit of 477 ppb is obtained if the integration time is extended to 265 s. According to the HITRAN database, the absorption coefficient of unit C_2_H_2_ at 6529.17 cm^−1^ is 0.618 cm^−1^ at room-temperature and atmospheric pressure. At an incident power of 30 mW and an integration time of 265 s, the NNEA of the system is calculated as α_min_·P·$\sqrt{t}$= 0.618·0.477 × 10^−6^·0.03·16.28 = 14.39 × 10^−8^ W·cm^−1^·Hz^−1/2^.

**Fig. 12:**
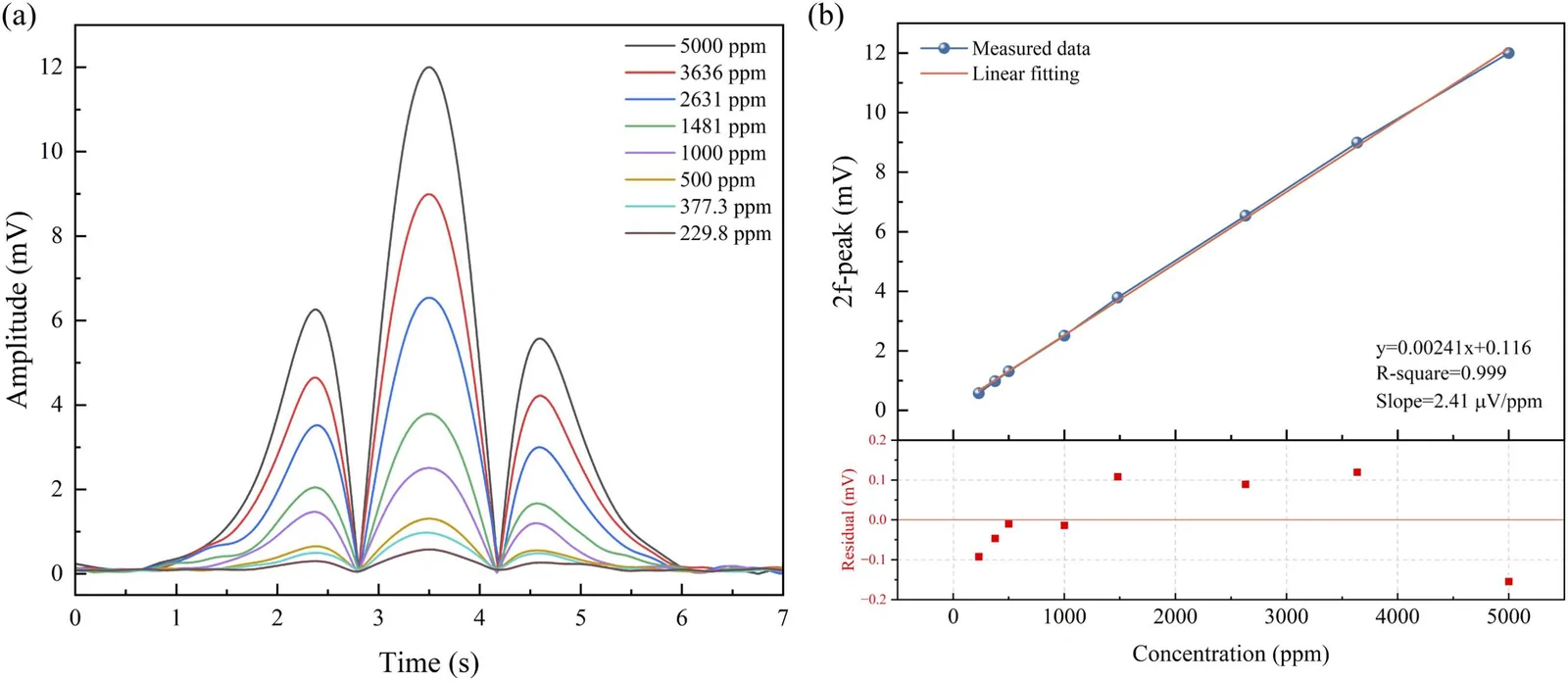
C_2_H_2_ concentration response of the MEMS cantilever-based PAS system. (a) Measured 2 f signals at C_2_H_2_ concentrations from 229.8 ppm to 5000 ppm. (b) Linear fitting of the extracted 2 f peak amplitude, with the corresponding residuals shown below.

**Fig. 13:**
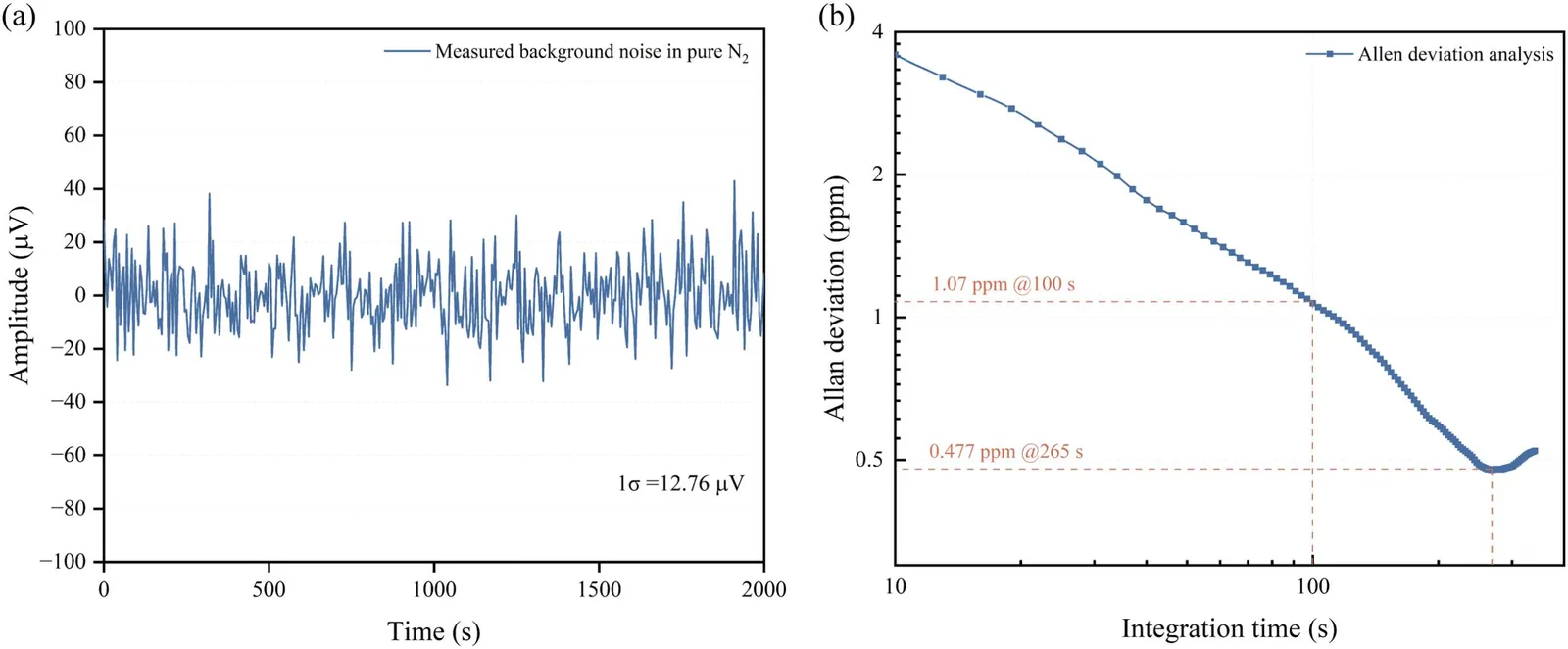
Background noise and detection limit analysis under pure N_2_. (a) Measured background amplitude over 0.5 h, with 1σ = 12.76 μV. (b) Allan deviation expressed as the equivalent C_2_H_2_ concentration, showing detection limits of 1.07 ppm at 100 s and 0.477 ppm at the integration time of 265 s.

## 4 Flexural-compliance-adjusted NNEA for cantilever-based PAS systems

The normalized noise equivalent absorption coefficient is widely used to evaluate the detection performance of photoacoustic gas sensors:

(1) $$NNEA=\alpha_{\min}P\sqrt{t}$$

where *α*_min_ represents the absorption coefficient at the minimum detectable concentration, *P* is the incident optical power, and *t* is the integration time. By accounting for the optical power and measurement bandwidth, conventional NNEA provides a practical system-level metric for comparing the detection sensitivity of different PAS configurations. In cantilever-based PAS systems, cantilever flexural compliance can contribute to acoustic-to-mechanical amplification. Long, thin, or low-rigidity structures can provide a larger mechanical response under a given acoustic pressure, potentially contributing to improved system-level sensitivity. However, conventional NNEA does not indicate the extent to which the reported performance is associated with this passive flexural-compliance advantage. Therefore, a complementary metric is introduced to account for this structural contribution. To quantify the passive flexural-compliance advantage arising from cantilever geometry and flexural rigidity, a flexural-compliance adjustment factor Ʌ is introduced in this work.

According to Euler–Bernoulli beam theory [40], the maximum free-end deflection *δ*_*m*_ of a cantilever beam under a uniformly distributed load *q* is:

(2) $$\delta_{m}=\frac{qL^{4}}{8{\left(EI\right)}_{eff}}=\frac{pwL^{4}}{8{\left(EI\right)}_{eff}}$$

where *L* is the cantilever length and (*EI)*_*eff*_ is the effective flexural rigidity. For a homogeneous rectangular cantilever, the effective flexural rigidity can be expressed as:

(3) $${\left(EI\right)}_{eff}=EI=E\frac{wt^{3}}{12}$$

where *E*, *w*, and *t* are the Young’s modulus, width, and thickness of the cantilever, respectively.

For a multilayer cantilever with the same width, the neutral axis *Z*_*NA*_ is first determined as:

(4) $$z_{NA}=\frac{\sum_{i}^{N}\frac{1}{2}E_{i}\left({z_{i}}^{2}-{z_{i-1}}^{2}\right)}{\sum_{i}^{N}E_{i}t_{i}}$$

where *t*_*i*_ is the thickness of each layer. *z*_*i-*1_ and *z*_*i*_ represent the coordinates of the lower and upper interfaces of the *i*th layer, respectively, measured from the reference surface. The effective flexural rigidity is subsequently calculated using composite-beam theory:

(5) $${\left(EI\right)}_{eff}=\sum_{i=1}^{N}\frac{1}{3}w_{i}E_{i}\left({\left(z_{i}-z_{NA}\right)}^{3}-{\left(z_{i-1}-z_{NA}\right)}^{3}\right)$$

where *E*_*i*_ and *w*_*i*_ are the Young’s modulus and width of each layer, respectively. For a uniformly applied acoustic pressure *p*, the equivalent line load is *q = pw*. The pressure-normalized deflection therefore can be expressed as:

(6) $$\frac{\delta_{m}}{p}=\frac{wL^{4}}{8{\left(EI\right)}_{eff}}$$

This term represents the structural pressure-to-deformation compliance of the cantilever. To express this compliance on a common stiffness scale, a reference Young’s modulus *E*_0_ is introduced, and the flexural-compliance adjustment factor Λ is defined as:

(7) $$\Lambda =E_{0}\frac{\delta_{m}}{p}=E_{0}\frac{wL^{4}}{8{\left(EI\right)}_{eff}}$$

In this work, *E*_0_ is set to 100 GPa. The reference Young’s modulus does not represent any material property, and the value is selected as a convenient characteristic stiffness scale within the range of commonly used solid materials for cantilever and MEMS structures. Because the same *E*_0_ is applied to all systems, changing its value uniformly scales all Λ and FCA-NNEA values and does not alter their relative ratios. When *E*_0_ is expressed in N/mm^2^, *L* and *w* are expressed in mm, and *(EI)*_*eff*_ is expressed in N·mm^2^, the unit of Λ is mm. The flexural-compliance adjustment factor Λ quantifies the extent to which the system can benefit from the passive flexural compliance of the cantilever, with a larger value indicating a greater contribution from compliance-induced mechanical amplification. The flexural-compliance-adjusted NNEA is then defined as:

(8) FCA-NNEA=NNEA·Λ

The unit of this new adjustment metric is cm^−1^·W·Hz^−1/2^·mm. It should be emphasized that Λ is intended to quantify the passive mechanical advantage provided by cantilever flexural compliance before the readout mechanism is considered. The subsequent conversion of the mechanical response into an optical or electrical signal remains specific to the individual readout scheme and is reflected in the experimentally measured conventional NNEA. FCA-NNEA therefore does not attempt to normalize different readout mechanisms but provides a structural-compliance-weighted measure of system performance. It could be regarded as a complementary metric rather than a universal ranking metric.

The comparison in Table 1 illustrates that the conventional NNEA and the flexural-compliance factor Λ describe different aspects of cantilever-based PAS performance. The factor Λ quantifies the passive flexural amplification available from the cantilever, with a larger value indicating a greater mechanical-compliance advantage. Several long and thin cantilevers combine low conventional NNEA with large Ʌ, indicating that their system-level performance is accompanied by substantial passive flexural amplification. After this compliance contribution is incorporated through FCA-NNEA, their performance no longer retains the same level of advantage. Conversely, several systems retain very low FCA-NNEA values even after flexural-compliance weighting. This indicates that their favorable system-level performance cannot be explained by high cantilever compliance alone but also reflect other effective elements of the complete PAS system, such as sensitive interferometric interrogation, acoustic enhancement, optimized transduction, and low-noise detection. These contributions remain embedded in the conventional NNEA and are not normalized by Λ.

**Table 1:** Quantitative comparison of representative cantilever-based PAS systems with sufficient structural information for FCA-NNEA evaluation.

Gas	Cantilever parameters	MDL	NNEA [cm^−1^·W·Hz^−1/2^]	Λ [mm]	FCA-NNEA [cm^−1^·W·Hz^−1/2^·mm]	Ref.
C_2_H_2_	*L*:6 mm *w*:1.5 mm *t*:10 μm	1.5 ppm @2.62 s 1.71 mW	3.6 × 10^−9^	1.15 × 10^9^	4.14	[41]
@1534.1 nm						
C_2_H_2_	*L*:2 mm *w*:0.8 mm *t*:6μm	35 ppb @100 s 7.8 mW	3.0 × 10^−9^	6.57 × 10^7^	0.197	[42]
@1532.8 nm						
CH_4_	*L*:2.3 mm *w*:1 mm *t*:10 μm	0.32 ppm @60 s ~20 mW	2.44 × 10^−8^	2.17 × 10^7^	0.531	[43]
@1650.9 nm						
H_2_S	*L*:5 mm *w*:1.2 mm *t*:10 μm	2.55 ppm @3.75 mW	5.1 × 10^−9^	5.36 × 10^8^	2.73	[44]
@1578 nm						
C_2_H_2_	*L*:1.039 mm *w*:0.25 mm *t*:6.43 μm	477 ppb @ 265 s 30 mW	14.39 × 10^−8^	5.97 × 10^6^	0.859	This work
@1531.6 nm						

^a^Only systems with sufficiently reported cantilever geometry, material information, and NNEA are included to calculate the flexural-compliance factor Λ and quantitative FCA-NNEA comparison.

The MEMS PZT cantilever in this work provides a different balance between structural compliance and system sensitivity. Although its conventional NNEA is not lower than those of several interferometrically readout systems, its FCA-NNEA remains within a competitive range with a comparatively small Λ, indicating that the achieved sensitivity is not primarily associated with an extremely compliant cantilever. Combined with its miniaturized dimensions and high piezoelectric coefficient, this result demonstrates that competitive PAS performance can be retained without relying on the large passive flexural amplification typically provided by long and thin cantilevers. This perspective is also consistent with recent piezoelectric cantilever PAS studies, in which performance enhancement increasingly relies on improved strain-to-electrical conversion through high piezoelectric coefficient materials, optimized active-layer and electrode configurations, and low-noise electrical readout. Representative PZT, PVDF, and PMN-PT-based PAS systems have demonstrated the potential of this approach, although insufficiently reported structural parameters presently prevent their inclusion in the quantitative FCA-NNEA comparison [38,39,45].

## 5 Conclusion

In this work, a microscale photoacoustic transducer with dimensions of 1039 μm × 250 μm × 6.43 μm was developed and successfully fabricated based on a multilayer MEMS cantilever. Coupled finite-element analysis predicted a 5124.7 Hz fundamental mode, with the simulated stress and induced charge density concentrated near the clamped region. The fabricated PZT cantilever exhibited a measured resonance at 5003.3 Hz with a 3-dB bandwidth of 33.74 Hz. The fabricated cantilever was integrated with an H-type photoacoustic cell designed for resonance-enhanced pressure generation and coupling. Using C_2_H_2_ as a representative target gas, the assembled system exhibited a resonance frequency of 4988 Hz. Under the optimized modulation conditions, the measured 2 f signal showed a linear response with a responsivity of 2.41 μV/ppm and an R^2^ of 0.999. Allan deviation analysis of the pure nitrogen background yielded a minimum detection limit of 477 ppb at an integration time of 265 s, corresponding to a conventional NNEA of 14.39 × 10^−8^ cm^−1^·W·Hz^−1/2^. These results confirm the effectiveness of the proposed MEMS PZT cantilever for acoustic-to-electrical conversion in resonant photoacoustic gas sensing.

To account for the influence of cantilever geometry and flexural rigidity on the evaluation of cantilever-based PAS systems, this work further proposed a flexural-compliance metric as an adjustment to the conventional NNEA. The resulting FCA-NNEA provides an additional basis for comparing cantilever transducers with substantially different dimensions and mechanical compliances. The comparison with representative reported systems indicates that the proposed device maintains competitive sensing performance with a cantilever footprint of only 0.26 mm^2^, substantially smaller than that of the millimeter-scale cantilevers used in many previously reported systems.

The present measurements were performed using dry gas mixtures under controlled laboratory conditions. For practical deployment, further evaluation under humid and multicomponent gas backgrounds will be required, since variations in gas composition and humidity may influence both spectral interference and collisional relaxation. Future work will therefore include calibration under representative interfering-gas and humidity conditions together with appropriate environmental compensation. In addition, customized MEMS cantilever arrays will be explored, in combination with multi-wavelength excitation, for parallel and selective detection in multi-gas environments. Further improvement of the sensor will also require optimization of the acoustic coupling, MEMS structure, and electrical readout. The pressure transfer efficiency could be increased by reducing volume and acoustic-impedance discontinuities between the resonant cavity and the cantilever, shortening the coupling path, and further optimizing the outlet geometry and sealing. From the MEMS perspective, rather than simply increasing cantilever compliance through a longer or thinner structure, locally narrowed or tapered geometries could be used to concentrate strain near the clamped region, together with optimized PZT and electrode placement in the high-stress region, thereby enhancing strain-to-charge conversion while retaining a compact size and suitable resonance frequency. Finally, a lower-noise charge-readout front end with reduced parasitic capacitance, optimized feedback components, and improved shielding could further reduce the electrical noise level.

## CRediT authorship contribution statement

**Shan Lin:** Writing – original draft, Methodology, Data curation, Conceptualization, Formal analysis, Validation. **Yongkang Gong:** Data curation. **Aron Michael:** Supervision, Methodology, Funding acquisition. **Fupeng Wang:** Supervision, Methodology. **Yinghang Jiao:** Data curation. **Gang-Ding Peng:** Supervision, Funding acquisition.

## Declaration of Competing Interest

The authors declare that they have no known competing financial interests or personal relationships that could have appeared to influence the work reported in this paper.

## Data Availability

Data will be made available on request.
